# *Culicoides hildebrandoi*, a new species of the reticulatus species group from the Brazilian Amazon Region (Diptera, Ceratopogonidae)

**DOI:** 10.3897/zookeys.571.7341

**Published:** 2016-03-07

**Authors:** Emanuelle de Sousa Farias, Antonio Marques Pereira Júnior, Maria Luiza Felippe-Bauer, Felipe Arley Costa Pessoa, Jansen Fernandes Medeiros, Maria Clara Alves Santarém

**Affiliations:** 1 Laboratório de Ecologia de Doenças Transmissíveis da Amazônia (EDTA), Centro de Pesquisa Leônidas e Maria Deane, Fiocruz Amazônia, Rua Terezina, 476, Adrianópolis, CEP 69057-070, Manaus, Amazonas, Brazil; 2 Fundação Universidade Federal de Rondônia, UNIR, Campus BR 364, Km 9.5, CEP 76801-059, Porto Velho, Rondônia, Brazil; 3 Laboratório de Entomologia, Fundação Oswaldo Cruz/Rondônia, Rua da Beira, 7671, Lagoa, CEP 76812-245, Porto Velho, Rondônia, Brazil; 4 Coleção de Ceratopogonidae, Laboratório de Diptera, Instituto Oswaldo Cruz-Fiocruz, Av. Brasil, 4365, 21040-900 Rio de Janeiro, RJ, Brazil

**Keywords:** Amazonas, biting midges, Brazil, Rondônia

## Abstract

A new species of biting midge (Diptera: Ceratopogonidae), *Culicoides hildebrandoi* **sp. n.**, is described and illustrated based on female and male specimens from the states of Amazonas and Rondônia, Brazil. This new species belongs to the *reticulatus* species group and differs from the 24 other species of this group by the elongate slightly swollen 3^rd^ palpal segment with scattered capitate sensilla but lacking a sensory pit.

## Introduction

The biting midges genus *Culicoides* Latreille (Diptera, Ceratopogonidae), presently includes 1355 extant worldwide species (Borkent 2015a), some of which can transmit pathogenic viruses and filarial nematodes to humans and other vertebrates. Due to their epidemiological importance, *Culicoides* are the best studied genus of Ceratopogonidae. Most species of *Culicoides* have wings with unique patterns of light and dark spots. Species with similar wing patterns have been included in subgenera or species groups with other similar morphological characters that are important for species identification (Felippe-Bauer 2003; Borkent 2015b).

The Neotropical *reticulatus* species group includes 24 species (Santarém et al. 2015). Santarém et al. (2014) redescribed *Culicoides reticulatus* Lutz and described seven new species, five of which were from three states in the Amazon Region of Brazil (Amazonas, Pará and Roraima). Santarém et al. (2014) noted an apparently undescribed, poorly mounted female from the state of Amazonas that they declined to describe. During our study of material from the state of Rondônia, we discovered an additional 12 female and four male specimens which we describe and illustrate herein.

## Materials and methods

Specimens were collected with CDC light traps in the forest on Rancho Colorado farm, Porto Velho municipality, state of Rondônia, Brazil. The specimens were preserved in 70% ethanol and subsequently slide-mounted in phenol-balsam with the methods described by Wirth and Marston (1968). The female specimen from Balbina, Amazonas studied by Santarém et al. (2014) was also examined. Diagnostic features were microphotographed using a Digital System (SynopticsTM, Cambridge, UK) adapted to an optical microscope (Leica DMTM1000, Frankfurt, Germany). Images were taken with a digital camera (JVC 3CCDTM, Wayne, USA), and Auto Montage 4.0 used to obtain the final images.

Morphological terms are from the chapter on Ceratopogonidae by Borkent et al. (2009), in the recent the Manual of Central American Diptera. Terms of structures specific to *Culicoides* follow those described by Felippe-Bauer (2003). Measurements of spermathecae are in micrometers, whereas those of wings are in millimeters. Meristic information is presented as ranges of values, followed by mean and sample size. The holotype, allotype and some paratypes were deposited in the Ceratopogonidae Collection of Oswaldo Cruz Foundantion (CCER), Rio de Janeiro, RJ, Brazil; other paratypes were deposited in the Laboratório de Ecologia de Doenças Transmissíveis da Amazônia, Leônidas and Maria Deane Institute (ILMD), Manaus, Amazonas, Brazil.

## Results

### Culicoides hildebrandoi sp. n.

Taxon classificationAnimaliaDipteraCeratopogonidae

http://zoobank.org/78B8970A-218C-4CBC-8388-AA4B248A11A2

Figs 1 , 2 , 3

#### Diagnosis.

Female: only species of *Culicoides* in the Neotropical Region with the following combination of features: 2^nd^ radial cell in dark spot, r_3_ with four sparsely distributed pale spots, r-m crossvein pale; hind femur with subapical pale band; scutum with two anterior submedian clover-leaf shaped spots; third palpal segment elongate, slightly swollen, cylindrical, without a sensory pit but with capitate sensilla scattered on the surface cuticle. Male: only species in the Neotropical Region with the following combination of features: tergite 9 with a posteromedial notch, parameres with slightly sinuous stem, swollen on mid-portion and without a ventral lobe and the basal arch of aedeagus extending 2/3 of total length.

#### Description.

**Female**. *Head* (Fig. 2d). Brown. Eyes bare, separated by distance equal to diameter of nearly one ommatidium (Fig. 2a). Antennal pedicel brown; flagellum pale brown, flagellomeres 1–8 pale on proximal ½; AR 0.90–1.00 (0.95, n = 8); sensilla coeloconica on flagellomeres 1, 6–8, two on 1, three on 6, three or four on 7 and four on 8. Palpus (Fig. 2b) brown; third segment elongate cylindrical, slightly swollen, without sensory pit, with sensilla scattered on surface; PR 3.30–3.90 (3.54, n = 12). Proboscis moderately long; P/H ratio 0.87–1.00 (0.95, n = 13); mandible (Fig. 2c) with 19–25 (22, n = 12) teeth.

**Figure 1.**
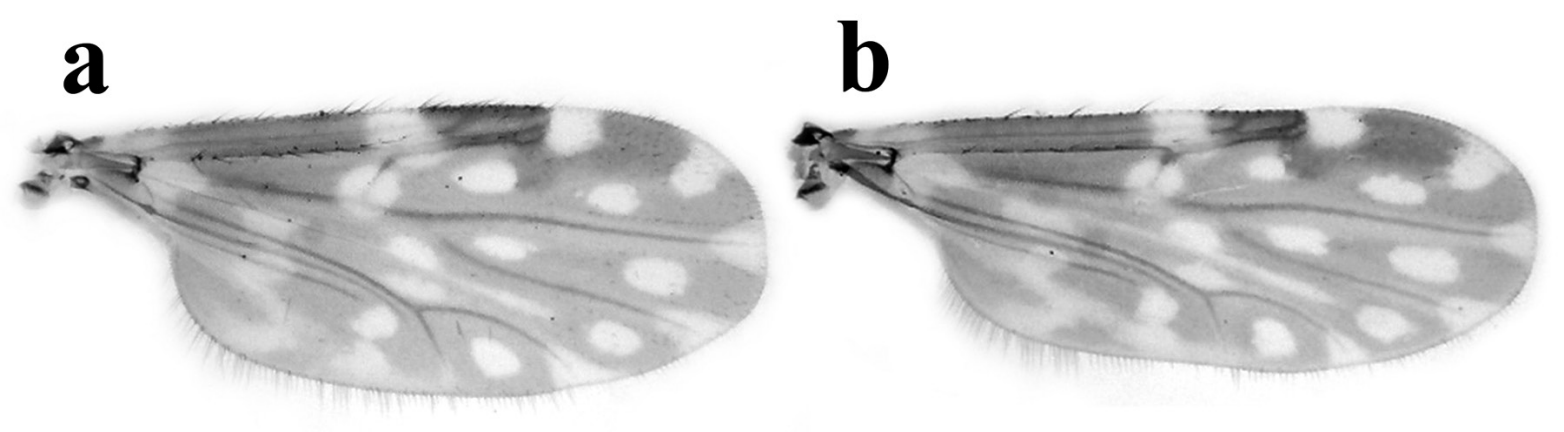
*Culicoides hildebrandoi* sp. n. Wing. **a** Female **b** Male.

**Figure 2.**
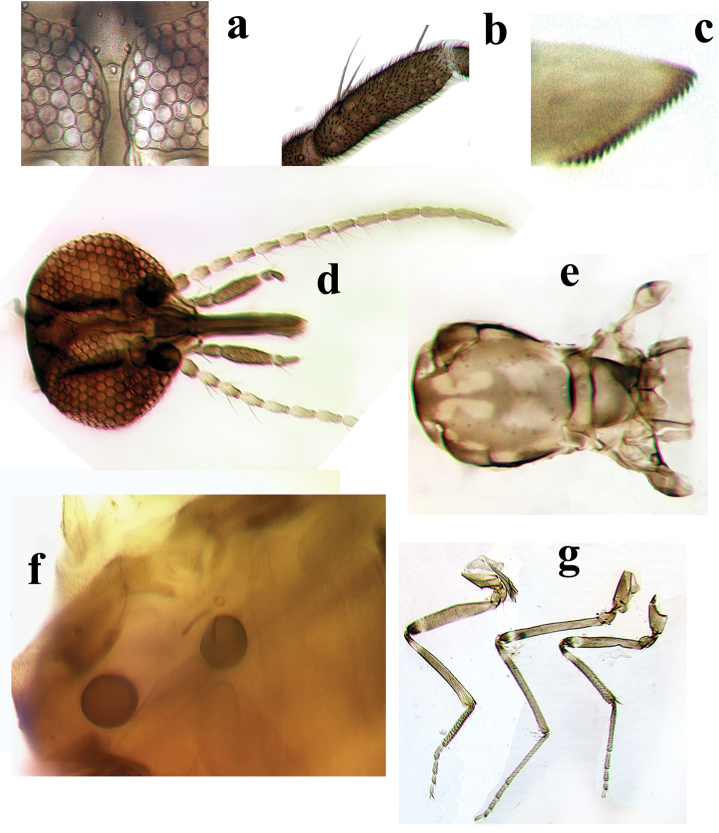
**a–g** *Culicoides hildebrandoi*, female sp. n. **a** Eye separation, anterior view **b** Palpal segment 3 **c** Mandibular teeth **d** Head, anterior view **e** Thorax, dorsal view **f** Spermathecae **g** Legs (right to left) fore-, mid- and hind.

*Thorax* (Fig. 2e). Dark brown, with prominent pattern of well-defined yellowish patches, humeral depression pale. Scutum with two anterior submedian clover-leaf shaped spots, two posterior submedian pale areas; lateral portion with anterior, posterior pale areas; prescutellar depressions pale; scutellum with yellowish lateral margins; postscutellum brown with pale median area. Wing (Fig. 1a) with contrasting pattern of pale and dark spots; distal ½ of 1^st^, all of 2^nd^ radial cell in dark spot; pale spot over r-m extending from M_1_ to just below radius; another pale spot from dorsal portion of radius to margin of costa; r_3_ with four small separated pale spots: 1^st^ rounded, between 2nd radial cell and M_1_; 2^nd^ extending posterior to 2nd radial cell, abutting wing margin; 3^rd^ ovoid, in mid portion of cell, larger than 2^nd^ spot; 4^th^ distal pale spot close to 3^rd^ spot, extending to wing margin; m_1_ with two pale spots, 1^st^ small, ovoid, beyond fork of M_1_ and M_2_, 2^nd^ larger than 1^st^, not close to wing margin; m_2_ with four pale spots: 1^st^ proximal to CuA, 2^nd^ and 3^rd^ between medial and mediocubital forks, 4^th^ larger, not reaching wing margin; cua_1_ with a rounded pale spot in middle of cell; anal cell with faint basal sinuous pale area and one distal pale spot near mediocubital fork that is nearly subdivided, abutting wing margin; wing base with faint pale spot on M; apices of M_1_, M_2_ and CuA_1_ broadly pale; macrotrichia sparsely distributed on distal half of wing; wing length 1.10–1.30 (1.25, n = 13) mm, breadth 0.50–0.58 (0.56, n = 12) mm; CR 0.60–0.68 (0.65, n = 13). Halter stem pale, knob brown. Legs (Fig. 2g) brown; femora with subapical, tibiae with subbasal pale bands; apex of hind tibia pale; hind tibial comb with four spines, that nearest spur longest.

*Abdomen*. Brown. Two subequal-size ovoid spermathecae (Fig. 2f), measuring 37.5–47.5 × 32.5–35.0 (n = 2) µm and 37.5–40.0 × 30.0–32.5 (n = 2) µm, slender sclerotized necks with 7.5 µm; third slender, elongate rudimentary spermatheca, length 27.5–30.0 (n = 2) µm.

**Figure 3.**
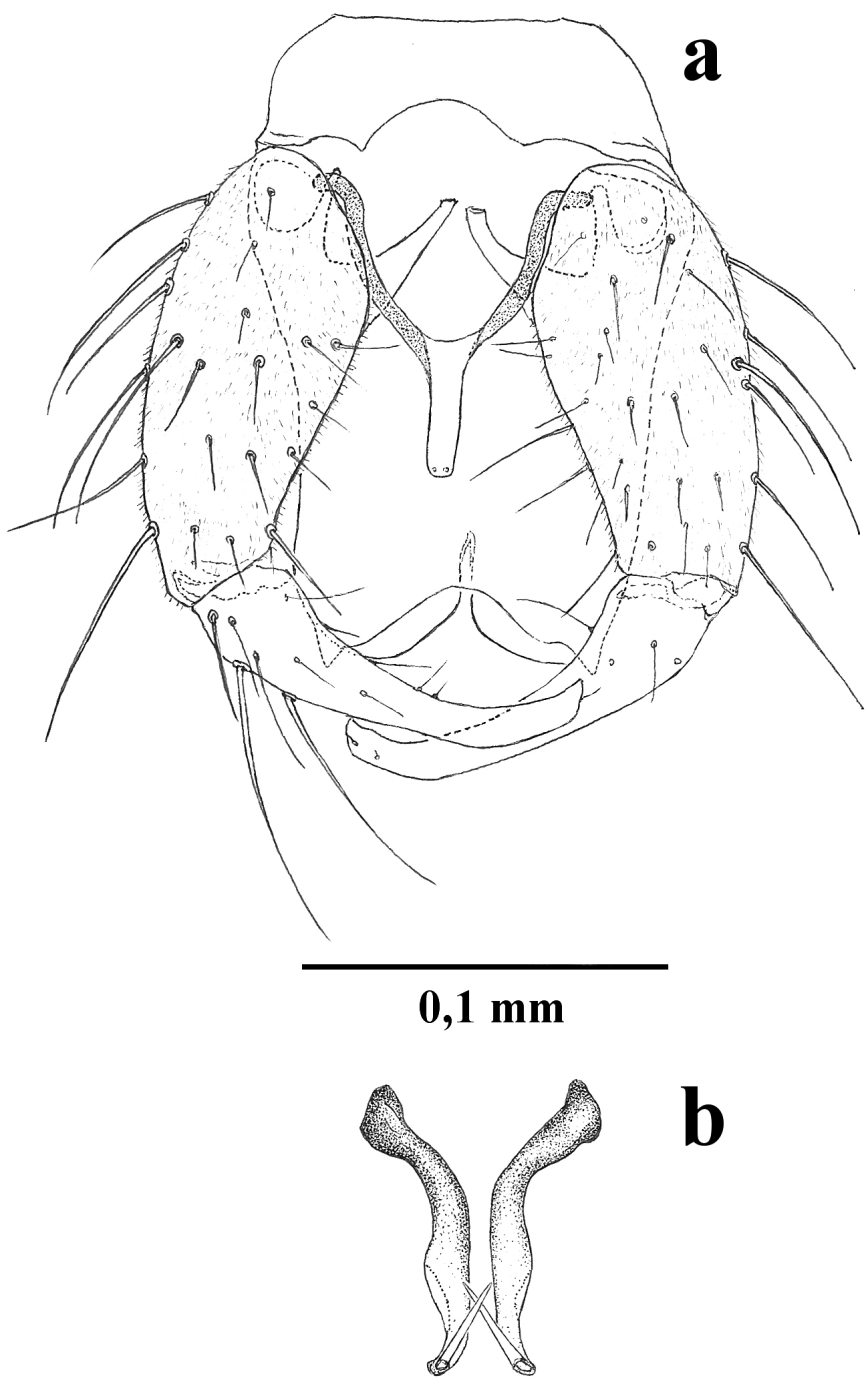
*Culicoides hildebrandoi*, male terminalia sp. n. **a** Terminalia and aedeagus (parameres removed), ventral view **b** Parameres, ventral view.

**Male.** Similar to female with usual sexual differences. Sensilla coeloconica on flagellomeres 1, 6-10, one on 1, 6-8, two on 9, three on 10; AR 0.80–0.87 (0.85, n = 4). PR 1.6–2.0 (1.78, n = 4). Wing with pattern of pale spots as in Fig. 1b, wing length 0.95–1.08 (1.00, n = 4), breadth 0.35–0.40 (0.38, n = 4); CR 0.62–0.64 (0.63, n = 4). Terminalia (Fig. 3a): Tergite 9 long, tapered slightly at mid length, distal portion broader with short conical apicolateral processes, with distinct posteromedial notch; sternite 9 with rounded posteromedian excavation. Gonocoxite twice as long as broad, ventral, dorsal roots slender, elongated, sclerotized; gonostylus tapering distally, distal portion curved, apex broader with beak-like tip. Parameres (Fig. 3b) separate, each one with heavily sclerotized basal knob; stem long, curved near base, slightly sinuous, swollen on mid portion without ventral lobe; apical portion tapered, elongate, abruptly bent without lateral fringe of spicules. Aedeagus (Fig. 3a) Y-shaped; basal arms heavily sclerotized; basal arch triangular, extending 2/3 of total length; distal portion moderately slender, apex rounded.

#### Type material.

Holotype female, labeled “Brazil, Rondônia, Porto Velho, Rancho Colorado Farm, 08°42'3.7"S; 63°59'3.8"W, 20.VIII.2014, CDC light trap, forest, Jansen F Medeiros col.” (CCER). Allotype male labeled as for female (CCER). Paratypes 12 females and three males: 11 females and three males same data as holotype (7 females, 2 males ILMD; 4 females, 1 male CCER); 1 female, Brazil, Amazonas, Balbina, 08.V.1985, CDC light trap, E. Castellón & S. Gomes col. (ILMD).

#### Distribution and bionomics.

This is a forest species of the Amazon region of Brazil in the states of Amazonas and Rondônia.

#### Etymology.

This species is named in memory of the late Dr. Luiz Hildebrando Pereira da Silva, a parasitologist who dedicated many years to studying Tropical Diseases such as Malaria and Chagas Disease. During his long career, Dr. Hildebrando was director of the Pasteur Institute, France. In Brazil he was a Medical School Professor at the University of São Paulo and Federal University of Rondônia. He also created the Institute for Research of Tropical Pathologies in Rondônia and was pivotal for the implementation of Oswaldo Cruz Foundation in Rondônia.

## Taxonomic discussion

*Culicoides hildebrandoi* sp. n. is very similar to *Culicoides reticulatus* and seven other closely related species described by Santarém et al. (2014) based on the number and distribution of pale spots on the wing and the clover-leaf pattern of yellowish patches on the anterio-submedian portion of the scutum. *Culicoides hildebrandoi* sp. n. can be distinguished from these eight congeners by its elongate, cylindrical third palpal segment with scattered capitate sensilla on its surface (third palpal segment is swollen and the capitate sensilla are in sensory pits in other species). Females of *Culicoides hildebrandoi* sp. n. are larger than related species with a wing length of 1.10–1.30 mm (wing length < 1.0 mm in *Culicoides amazonicus* Santarém, Felippe-Bauer & Trindade, *Culicoides diplus* Santarém & Felippe-Bauer, *Culicoides profundus* Santarém, Felippe-Bauer & Trindade, *Culicoides pseudoreticulatus* Santarém, Felippe-Bauer & Castellón and *Culicoides rhombus* Santarém, Felippe-Bauer & Castellón), they have a more slender 3^rd^ palpal segment, PR 3.3–3.9 (PR < 3.2 in the other species) and, a moderately long proboscis, P/H ratio 0.87–1.00 (P/H ratio ≥ 1.00 in *Culicoides amazonicus*, *Culicoides diplus*, *Culicoides fluminensis* Santarém & Felippe-Bauer, *Culicoides pseudoreticulatus*, *Culicoides reticulatus* and *Culicoides rhombus*).

This new species along with *Culicoides amazonicus*, *Culicoides irregularis* Santarém, Felippe-Bauer & Castellón, *Culicoides profundus*, *Culicoides pseudoreticulatus* and *Culicoides rhombus* are associated with forested environments in the Brazilian Amazon Region, while the other three congeners are associated with mangrove swamps in the coastal regions of Colombia and Panama (*Culicoides diplus*), Rio de Janeiro, Brazil (*Culicoides fluminensis*), and Bahia and Pernambuco, Brazil (*Culicoides reticulatus*).

With the description of *Culicoides hildebrandoi* sp. n. here the “*reticulatus* species group” now has 25 species distributed throughout the Neotropics.

## Supplementary Material

XML Treatment for Culicoides hildebrandoi
